# Long Noncoding RNAs Regulate the Radioresistance of Breast Cancer

**DOI:** 10.1155/2021/9005073

**Published:** 2021-09-20

**Authors:** Zhifeng Li, Fujin Wang, Yinxing Zhu, Ting Guo, Mei Lin

**Affiliations:** ^1^Department of Oncology, Medical College of Nantong University, Nantong, 226001 Jiangsu, China; ^2^Department of Radiology, The First People's Hospital of Yancheng, Yancheng, 224006 Jiangsu, China; ^3^Taizhou People's Hospital Affiliated to Nanjing University of Chinese Medicine, Taizhou, 225300 Jiangsu Province, China; ^4^Research Center of Clinical Medicine, Taizhou People's Hospital (the Affiliated Hospital 5 of Nantong University), Taizhou, 225300 Jiangsu Province, China

## Abstract

Breast cancer (BRCA) has severely threatened women's health worldwide. Radiotherapy is a treatment for BRCA, which applies high doses of ionizing radiation to induce cancer cell death and reduce disease recurrence. Radioresistance is one of the most important elements that affect the therapeutic efficacy of radiotherapy. Long noncoding RNAs (lncRNAs) are suggested to dominate crucial roles in regulating the biological behavior of BRCA. Currently, some studies indicate that overexpression or inhibition of lncRNAs can greatly alter the radioresistance of BRCA. In this review, we summarized the knowledge on the classification and function of lncRNAs and the molecular mechanism of BRCA radioresistance, listed lncRNAs related to the BRCA radioresistance, highlighted their underlying mechanisms, and discussed the potential application of these lncRNAs in regulating BRCA radioresistance.

## 1. Introduction

Breast cancer (BRCA) is the most common cancer that affects women's health worldwide. According to the International Agency for Research on Cancer (IARC), BRCA has the highest incidence among all cancers globally. The treatment plan for BRCA depends greatly on its molecular subtypes, which can be categorized into four major classes: luminal A, luminal B, HER-positive, and triple-negative breast cancer (TNBC) [1]. Currently, five standard therapeutic options, namely, surgery, chemotherapy, hormonal therapy, targeted therapy, and radiotherapy, are available for BRCA [2, 3]. Radiotherapy (RT) accounts for an important part of the comprehensive treatment for BRCA, especially in the treatment of advanced breast cancer (ABC) [4], metastatic breast cancer (MBC) [5], triple negative breast cancer (TNBC) [6], and breast-conserving surgery [7]. Based on our knowledge, the therapeutic efficacy of RT is usually related to tumor radiosensitivity, which refers to the injury or damage speed of cells, tissues, and organs exposed to radiation [8]. RT has brought great benefits for the treatment of BRCA. However, based on results from clinical observation, some patients develop tumor recurrence shortly after RT, and radioresistance appears to be the bottleneck of RT for BRCA. Therefore, how to reduce radioresistance and enhance the radiosensitivity of BRCA has become an urgent problem for researchers. Several years ago, researchers saw a glimmer of hope from long noncoding RNAs (lncRNAs). lncRNA is a nonprotein encoding nucleotide sequence that is over 200 nucleotides in length. The biological functions of lncRNAs mainly include the regulation of gene methylation [9], activation of transcription, regulation of cell cycle, DNA damage repair, and modulation of mRNA translation [10]. Based on the complex biological function, numerous researchers have explored the relationships of lncRNAs with radioresistance and achieved some encouraging results. Recently, although many studies have shown that a certain number of lncRNAs play important roles in regulating BRCA radioresistance, none of them has yet made a comprehensive and systematic summary of these lncRNAs. Herein, we searched relevant human studies and English language publications available in the PubMed, Scopus, and Web of Science. Both Medical Subject Headings (MeSH) terms and related free words were used to increase the sensitivity of the search. In addition, we reviewed the classification and functions of lncRNAs, summarized the radioresistance mechanisms of cancer cell, and listed the lncRNAs confirmed to regulate the BRCA radioresistance, aiming to provide better understanding for coming researchers.

## 2. Overview of lncRNAs

lncRNAs were first described at around 1990 [11], while they did not attract much attention from scholars at that time. Originally, lncRNAs were regarded as the “noises” of genome transcription, or some by-products of RNA polymerase II transcription. The first widely studied lncRNA was H19 [12, 13], and it was detected in various types of tumor cells [14]. Currently, a definition of lncRNAs based on their length and function is widely accepted. To be specific, lncRNAs are a group of nucleotide sequences with similar structure to mRNA and are more than 200 nucleotides in length, which cannot encode functional proteins due to the lack of a canonical open reading frame (ORF) [15, 16]. Since the 21st century, with the development of gene sequencing technology, an increasing number of lncRNAs have been found. Moreover, genome-wide association studies on tumor samples have identified numerous lncRNAs associated with various types of cancers, including BRCA, ovarian cancer, and hepatocellular carcinoma (HCC) [17, 18]. Besides, several lncRNAs have been reported to play crucial roles in diverse biological processes, including cell proliferation, apoptosis, metastasis, invasion, metastasis, differentiation, chromatin modification, and nuclear-cytoplasmic trafficking [19, 20]. lncRNAs are pervasively transcribed throughout the genome, and the resulting transcripts are remarkably similar to the classical mRNAs [21], but the origin of lncRNAs has not been clearly described. Generally, there are five possibilities for their origin, namely, the interruption and transformation of protein-coding genes, the formation of chromosome recombination, the formation of reverse transcription of noncoding genes in the process of replications, the formation of tandem adjacent replicon, and the insertion of transposable elements into genes [22].

Although different scholars have made many categories of lncRNAs according to different classification methods [23–26], there are still some newly discovered lncRNAs that cannot be attributed to these types. It appears that these classification methods are still not comprehensive enough [27]. At present, the most commonly used and relatively comprehensive way of category relies on lncRNAs' function, structure, and biogenesis. Specifically, based on function, lncRNAs can be classified into *cis*-lncRNA, ceRNA, and *trans*-lncRNA; according to structure, they may be sorted as circular RNA and linear RNA including four subclasses (lincRNA, eRNA, TUCRNA, and NAT); in terms of biogenesis, lncRNAs can be classified into intronic RNA, enhancer RNA, promoter RNA, antisense RNA, sense RNA, intergenic RNA, and bidirectional RNA (Table 1).

Over the past few decades, especially the last decade, the functions of numerous lncRNAs in different tissues and diseases have been elucidated. lncRNAs have very complex biological functions, including (1) interference with the target gene transcription into mRNA; (2) mediation of chromatin remodeling and histone modification to regulate the balance between euchromatin and heterochromatin; (3) interference with the mRNA splicing sequence; (4) formation of interfering RNA that binds to the target mRNA and leads to its degradation; (5) changes of protein structure and function and regulation of protein activity; (6) regulation of protein activity as miRNAs, such as small interfering RNAs (siRNAs) and the miRNA precursors, which play a role of endogenous “miRNA sponge”, inactivate miRNAs by adsorption, affect the competitive endogenous RNA (ceRNA) network, and ultimately affect the target gene expression of miRNA; (7) alteration of protein localization in the cells; and (8) production of small RNAs, such as miRNAs, piRNAs, and other less well-characterized classes of small transcripts [28] .

## 3. Mechanism of Breast Cancer Radiotherapy

**Figure 1:**
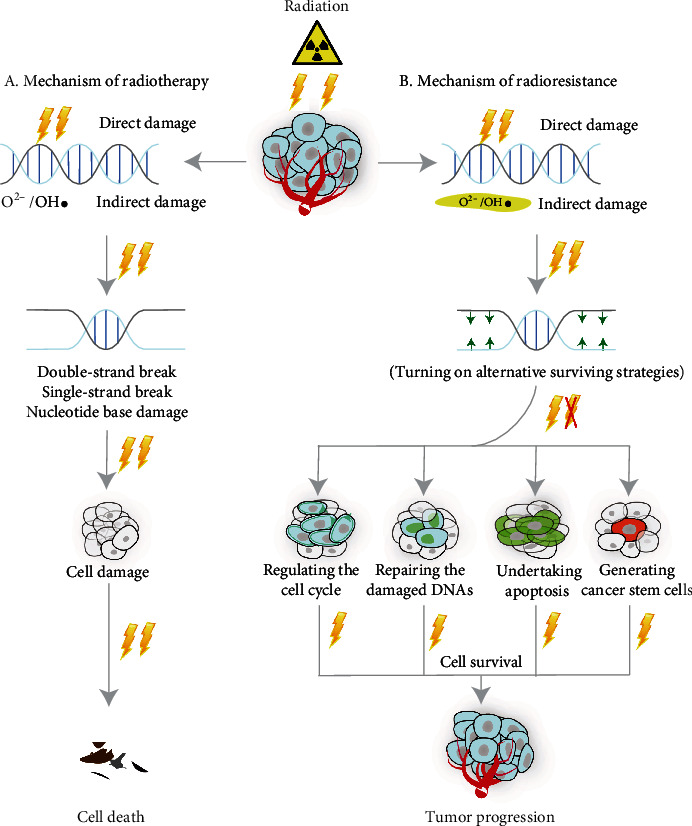
Mechanism of action of radiotherapy and development of radioresistance. (a) (1) Mechanisms of action of radiotherapy. The principle of RT is classified into two types according to different injury mechanisms: direct effect and indirect effect. (2). DNA damage: double-strand break, single-strand break, and nucleotide base damage. (b) Major mechanisms of radioresistance: (1) regulating the cell cycle, (2) repairing the damaged DNAs, (3) escaping from apoptosis, and (4) generating the CSCs.

The basis of radiotherapy is to use radiation or ionization energy to damage molecular structures and then kill cancer cells [29]. The principle of RT can be classified into two types according to different injury mechanisms: (1) The first one is the direct effect of RT, where radiation or ionization energy directly damages molecular structures in cells such as DNA [30]. (2) The second one is the indirect effect of RT. During the release of radiation energy, water molecules in both the cellular and extracellular environments can be attacked by radiation [31] to generate the highly reactive and unstable reactive oxygen species (ROS), like superoxide (O^2-^) and hydroxyl radicals (OH) [30, 32]. Then, cells are damaged by means of oxidative stress (OS). Both the direct and indirect effects of RT may cause DNA damage, including double-strand break (DSB), single-strand break (SSB), and nucleotide base damage [32](Figure 1(a)). RT in BRCA is indicated mainly in the postoperative context (adjuvant) to eliminate the microscopic disease. In many of the published mainstream clinical guidelines for BRCA management, RT is always emphasized as one of the key treatment options and the fundamental part of the multidisciplinary management of BRCA [33–35].

There are generally two types of radiation in the treatment of BRCA. One is external beam radiation, which involves the delivery of high-energy radiation beams from outside the body. The other one is brachytherapy, which involves the placement of a radiation-emitting substance near BRCA tissues over a certain period of time to kill the tumor [36]. However, radioresistance may arise when the irradiated cancer cells turn on the alternative mechanisms to promote their survival, proliferation, and invasion and escape from cell death, either in external beam radiation or brachytherapy [37]. It is true that RT has become one of the most important treatment options for the comprehensive treatment of BRCA, while its efficacy remains limited and plagued by the development of radioresistance [38, 39].

## 4. Cellular Mechanisms of Cancer Radioresistance

The irradiated cancer cells may react in several ways to the arising radioresistance, such as regulating the cell cycle, repairing the damaged DNAs, or undertaking apoptosis/autophagy [40]. Another cellular mechanism that can promote radioresistance is to generate cancer stem cells (CSCs) [41]. We elaborate on the above mechanism as follows and summarize in a figure (Figure 1(b)). Cellular mechanisms of cancer radioresistance are as follows: (1) regulating the cell cycle: the purposes of cell cycle checkpoints are to provide surveillance and prevent abnormal cells from actively dividing and temporarily blocking the cell cycle, so as to give time for DNA repair [42]. Some critical regulatory proteins such as ATF3 [43] and Rac1 [44, 45] are reported to be involved in the regulation of BRCA radioresistance. (2) Repairing the damaged DNAs: there are a few mechanisms by which DNA damage may be repaired postirradiation, namely, homologous recombination (HR) [46], nonhomologous end joining (NHEJ) [47], single-strand break repair (SSBR) [46, 48], and base excision repair (BER) [49]. (3) Escaping from apoptosis/cellular suicide: in the event where DNA is severely damaged to the extent that is hard to be repaired, expression of apoptotic-related genes may increase, while escape from apoptosis is another way for cancer cells to achieve radioresistance [50]. Several studies report that some apoptotic-related proteins such as hypoxia-inducible factor-1(HIF-1*α*), C/EBP homologous protein (CHOP), and c-Jun NH2-terminal kinase (JNK) are connected to radioresistance in BRCA cells [51, 52]. Besides, the switching on and activation of various oncogenes that promote cellular proliferation, invasion, and metastases such as HRAF, TNF, PBK, AKT1, ZNF304, ZNF611, NF-jb, Snail, and E-cadherin [53] have also been associated with the promotion of radioresistance in BRCA cells. (4) Regulating or generating the CSCs: the radioresistance of cancer cells is related to the number of CSCs. As a rule, the number of CSCs may increase after radiotherapy. An explanation for the emergence or increase of CSCs postirradiation is that radiation kills radiosensitive cancer cells and allows the intrinsic radioresistant CSCs to propagate. In another study [54], radiation is shown to induce the development of breast CSCs from non-CSCs.

## 5. lncRNAs in Regulating the Radioresistance of Breast Cancer

**Figure 2:**
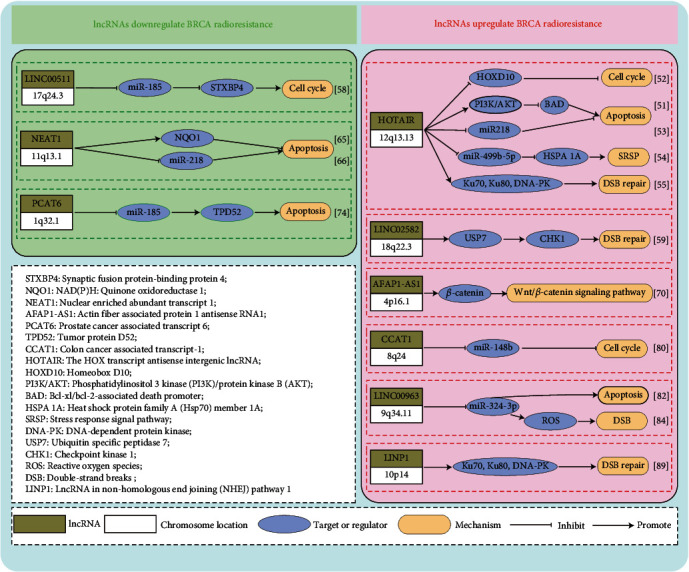
lncRNAs downregulate or upregulate BRCA radioresistance via different roles. Major mechanisms regulating the tumor radioresistance of lncRNAs: (1) DNA double-strand repair, (2) IR-induced apoptosis, (3) IR-induced autophagy, (4) cell cycle, and (5) others: glycolysis, the Wnt/*β*-catenin pathway, SRSP.

lncRNAs form complex secondary structures and are highly regulated. By the protein and nucleic acid-binding abilities, lncNRAs can modulate protein-protein and protein–nucleic acid interactions that control many cellular processes. According to existing studies, lncRNAs may regulate tumor radioresistance through the following mechanisms, including DNA double-strand repair [55, 56], IR-induced apoptosis [57], IR-induced autophagy [58], cell cycle [59], and the Wnt/*β*-catenin pathway [60, 61]. Are the diverse functions of lncRNAs a mirage in regulating BRCA radioresistance? Thanks to researchers who pay attention to the correlation between lncRNAs and BRCA radioresistance, some abnormally expressed lncRNAs that regulate the radioresisitance of BRCA have been studied, and the related mechanisms have been preliminarily expounded at the molecular level. Recently, related findings have been gradually reported. Therefore, it is now known that some lncRNAs can enhance radioresistance, whereas others may reduce radioresistance. This study systematically summarized the functional lncRNAs involved in regulating the radioresistance of BRCA reviewed the current research on lncRNAs regulating the radioresisitance of BRCA (Figure 2) and elaborated their possible mechanisms separately as follows.

### 5.1. The HOX Transcript Antisense Intergenic lncRNA (HOTAIR)

HOX transcript antisense intergenic lncRNA (HOTAIR) is located in the homeobox C (HOXC) gene cluster between the HOXC11 and HOXC12 genes on human chromosome 12q13.13, which is expressed at the HOXC gene locus [62]. HOTAIR has been well demonstrated to be positively correlated with metastasis, chemoresistance, and malignancy of BRCA [63]. Recently, some studies suggest that HOTAIR negatively regulates the radiosensitivity of BRCA cells. Zhou et al. [64] examined the expression of HOTAIR gene in five BRCA cell lines, discovering that the upregulation of HOTAIR in MDA-MB231 cells accelerated cell proliferation and enhanced the radioresistance. The expression levels of HOXD10 (whose translation is repressed by HOTAIR) [65], pBAD (BRCAl2-associated agonist of cell death that is involved in the apoptotic pathway), and pAKT (involved in the cell proliferation pathway) are evaluated to investigate the mechanism in controlling the HOTAIR-induced radioresistance. The results show that HOTAIR promotes the proliferation of BRCA cells during RT by targeting HOXD10 and the PI3K/AKT-BAD pathway [64]. Hu et al. [66] confirmed that HOTAIR was upregulated in BRCA cells and tissues, and its expression increased after radiation. Besides, knockdown of HOTAIR induced DNA damage, inhibits cell survival, and increases cell apoptosis in response to ionizing radiation (IR). Further experiments show that the radiosensitization effect of HOTAIR is related to the upregulation of miR-218, a ceRNA of HOTAIR involved in the apoptosis and in repair of radiation-induced DNA damage [63]. Zhang et al. [67] confirmed that the overexpression of HOTAIR significantly enhanced the proliferation of MDA-MB-231 and MCF-7 cells in a time-dependent manner after exposure to the 10 Gy gamma radiation. Further experiments indicate that lncRNA HOTAIR alleviates the miR-449b-5p-mediated inhibition of heat shock protein family A (Hsp70) member 1A (HSPA1A) by acting as a molecular sponge targeting miR-449b-5p and enhances HSPA1a expression in the stress response signal pathway (SRSP) to enhance the radioresistance of BRCA. Besides, HOTAIR is upregulated in invasive ductal carcinoma of BRCA, which can promote the proliferation of BRCA cells by regulating the cell cycle and apoptosis. Qian et al. [68] found that knockdown of HOTAIR expression significantly reduced the survival rate of MCF7 cells after 4 Gy radiation, while upregulation of HOTAIR expression improved the survival rate. Moreover, upregulation of HOTAIR promotes the expression of Ku70, Ku80, DNA-dependent protein kinase catalytic subunit (DNA-PKs), and ATM in the DNA double-strand break repair pathway, thus reducing the radiosensitivity of BRCA cells.

### 5.2. Long Intergenic Noncoding RNA 00511 (LINC00511)

Located on chromosome 17q24.3, long intergenic noncoding RNA 00511 (LINC00511) is significantly upregulated in BRCA, and its expression level is correlated with the poor prognosis of BRCA, such as cancer cell proliferation, invasion, and chemoresistance [69, 70]. Liu et al. [71] detected LINC00511 expression in BRCA tissues and cell lines. They found that LINC00511 expression was significantly upregulated, which was correlated with recurrence and poor survival after breast-conserving surgery followed by RT. Further research confirms that LINC00511 knockdown by shRNA restricts cell proliferation, promotes cell apoptosis, enhances radiosensitivity in vitro, and inhibits tumor growth, with an increased response to radiation in vivo. Upregulation of LINC00511 increases the expression of synaptic fusion protein-binding protein 4 (STXBP4) through competitive binding with miR-185, while silencing LINC00511 weakens its competitive binding with miR-185 and decreases the expression of STXBP4. Moreover, evolutionary studies have concluded that LINC00511 reduces the radioresistance of BRCA via the LINC00511/miR-185/STXBP4 axis.

### 5.3. Long Intergenic Noncoding RNA 02582 (LINC02582)

Long intergenic noncoding RNA (LINC02582) is located on chromosome 18q22.3. Over the past, little is known about the function of linc02582 in tumors. Recently, Wang et al. [72] conducted some pioneering preliminary research on this aspect. They found that LINC02582 specifically bound to ubiquitin-specific peptidase 7 (USP7) to inhibit the deubiquitination of cell cycle checkpoint kinase 1 (CHK1), which thus stabilized CHK1 protein, and improved the CHK1 protein level and radioresistance of BRCA cells. In addition, inhibition of LINC02582 expression enhanced radiosensitivity, while overexpression of LINC02582 promoted radioresistance of BRCA cells both in vitro and in vivo.

### 5.4. Nuclear-Enriched Abundant Transcript 1 (NEAT1)

Nuclear-enriched abundant transcript 1 (NEAT1), also called nuclear paraspeckle assembly transcript 1, was first discovered in 2007 [73]. It is located on chromosome 11q13.1 and can be divided into two subtypes, namely, NEAT1-1 and NEAT-2, which differ in the processing of their 3′ untranslated region (UTR). NEAT1 does not encode proteins in the nucleus, but it generates the core structural component of “speckle” subcellular organelles, which plays an important component in the development and progression of BRCA [74, 75]. For example, the aberrant paraspeckle formation in BRCA may result in the higher resistance to apoptosis induced by chemotherapy or RT [76, 77]. Recently, Lin et al. [78] found that lncRNA NEAT1 positively regulated the expression of NAD(P)H: quinone oxidoreductase 1 (NQO1) in radioresistant MDA-MB-231 cells at the translation level. Further, NQO1 knockdown inhibited the proliferation of radioresistant MDA-MB-231 cells, whereas NOQ1 overexpression increased their survival. Moreover, inhibition of NEAT1 expression by the CRISPR-Cas9 method enhanced the sensitivity of radioresistant MDA-MB-231 cells to radiation and reduced their proliferation. The authors further suggested that NEAT1 regulated BRCA radioresistance via NOQ1. Besides, another study shows that NEAT1 negatively regulates miR-218 expression and promotes BRCA progression [79]. As we know, miR-218 is involved in the repair of radiation-induced DNA damage and in apoptosis [63]. Therefore, NEAT1 may also adjust the BRCA radioresistance via the miR-218 pathway.

### 5.5. Actin Filament-Associated Protein 1 Antisense RNA1 (AFAP1-AS1)

Actin fiber-associated protein 1 antisense RNA1 (AFAP1-AS1), located on chromosome 4p16.1, is a well-known lncRNA overexpressed in various tumor tissues and cell lines [80, 81]. Upregulation of AFAP1-AS1 promotes BRCA cell proliferation and invasion but inhibits cell apoptosis in vitro, while overexpression of AFAP1-AS1 promotes tumor growth in vivo and is associated with poor prognosis of TNBC patients [82]. Bi et al. [83] selected AFAP1-AS1 from the locally recurrent BRCA samples after RT and the radioresistant BRCA cell line MDA-MB-231. They discovered that lncAFAP1-AS1 was not only upregulated in radioresistant cells and tumor tissues but was also associated with a low survival rate of BRCA patients from the TCGA database. Additionally, lncAFAP1-AS1 expression in tumor tissues from locally recurrent BRCA patients was significantly higher than that in patients without recurrence. The authors further confirmed that the high expression of lncAFAP1-AS1 was related to radioresistance of BRCA patients, and lncAFAP1-AS1 expression in tumor tissues might be used to predict the outcome of RT for BRCA. In addition, the authors proved that lncAFAP1-AS1 blocked the function of *β*-catenin destruction complex, which then led to the release of *β*-catenin and the activation of the Wnt/*β*-catenin signaling pathway, thus inducing the radioresistance of BRCA.

### 5.6. Prostate Cancer-Associated Transcript 6 (PCAT6)

Prostate cancer-associated transcript 6 (PCAT6), located on chromosome 1q32.1, is upregulated in various human malignancies, including lung cancer, HCC, cervical cancer, and BRCA [84, 85]. It can promote tumorigenesis and angiogenesis of TNBC by regulating the epidermal growth factor receptor (EGFR) [86]. Recently, Shi et al. [87] found that PCAT6 and tumor protein D52 (TPD52) were highly expressed in BRCA tissues and cells, while miR-185-5p was lowly expressed. PCAT6 directly interacts with miR-185-5p and negatively regulates the expression of miR-185-5p, thereby regulating TPD52, the direct target of miR-185-5p. PCAT6 can also regulate the expression of TPD52 by acting as the molecular sponge of miR-185-5p. Knockdown of PCAT6 reduces cell proliferation and promotes cell apoptosis by upregulating the expression of miR-185-5p and downregulating that of TPD52, thus promoting the radiosensitivity of BRCA cells. This may indicate that PCAT6 regulates the radioresistance of BRCA via the PCAT6/miR-185-5p/TPD52 signaling pathway.

### 5.7. Colon Cancer-Associated Transcript-1 (CCAT1)

Colon cancer-associated transcript-1 (CCAT1) is a highly specific molecular marker for CRC, which is transcribed from chromosome 8q24 with a length of 2600 nucleotides. Some studies have revealed that CCAT1 is highly expressed and plays an oncogenic role in different types of cancers. Its aberrant expression is involved in tumorigenesis, progression, metastasis, and patient survival through regulating different target genes and signaling pathways [88–90]. Recently, CCAT1 is also found to show high expression in BRCA tumors and cells, which promotes the proliferation, migration, and invasion of BRCA cells by targeting the miR-218/ZFX axis [91], enhances the function of BRCA stem cells by activating the Wnt/*β*-catenin signaling pathway [92], and can serve as a biomarker for predicting the prognosis of BRCA. Compared with radiosensitive BRCA, CCAT1 is upregulated whereas miR-148b is downregulated in radioresistant BRCA. RT triggers the significantly increased expression of CCAT1 but the remarkably decreased expression of miR-148b. The downregulation of CCAT1 results in the upregulation of miR-148b, which decreases the colony formation ability and caspase-3 activity in BRCA cells. Besides, it is elucidated in a study that upregulation of CCAT1 enhances the radioresistance of BRCA cells by negatively regulating miR-148b expression [93].

### 5.8. Long Intergenic Noncoding RNA00963 (LINC00963)

Located on chromosome 9q34.11, long intergenic noncoding RNA00963 (LINC00963) is overexpressed in BRCA specimens and plays an important role in the biological processes of BRCA [94]. Silencing of LINC00963 inhibits the proliferation and tumorigenesis of BRCA cells, while the overexpression of LINC0963 has opposite effects. LINC00963 affects the proliferation, migration, invasion, and apoptosis of BRCA cells in various ways, which is related to lymph node metastasis (LNM), TNM stage, and differentiation degree [95]. Besides, LINC00963 is negatively correlated with the expression of small noncoding miR-324-3p in BRCA tissues. According to Kuo et al. [96], overexpression of miR-324-3p inhibited the growth and invasion of BRCA cells. The radiation-induced ROS production is enhanced upon the overexpression of miR-324-3p. Knockdown of LINC00963 increases the expression of miR-324-3p, leads to DNA damage and OS of ROS, and reduces the radioresistance of BRCA cells [97].

### 5.9. lncRNA in Nonhomologous End Joining (NHEJ) Pathway 1 (LINP1)

Located on chromosome 10p14, lncRNA in nonhomologous end joining pathway 1 (LINP1) is a lncRNA associated with the nonhomologous end joining (NHEJ) repair pathway 1 [98]. It is well known that the repair of double-strand breaks (DSBs) in DNA by the NHEJ pathway is one of the main ways for tumor cells to repair the damaged DNA after RT and chemotherapy [99]. NHEJ is initiated when a DNA DSB is detected and bound to the Ku70–Ku80 heterodimer. Ku then recruits additional NHEJ proteins to the DSBs, which process and subsequently ligate the broken DNA ends. One well-characterized Ku-interacting protein is the DNA-PKCs, a serine/threonine-protein kinase essential for NHEJ in human cells. LINP1 shows higher expression in BRCA tissues than in adjacent noncarcinoma tissues [100] and interacts with two proteins, namely, Ku80–Ku70 heterodimer and DNA-PKCs, which play important roles in the NHEJ process after RT. Specifically, it acts as a scaffold that connects Ku80 with DNA PKCs to enhance the repair of DNA DSBs [101], thus coordinating the NHEJ pathway. Zhang et al. [102] demonstrated that LINP1 interacted with and enhanced the formation of the Ku–DNA–PKCs complex. Moreover, depletion of LINP1 attenuates DSB repair, induces radiation sensitivity, and delays tumor growth in mouse xenografts after exposure to IR. Together, their data reveal that the interaction of LINP1 with the Ku–DNA–PKCs complex enhances the NHEJ-mediated DSB repair and that depleting LINP1 downregulates the radioresistance of BRCA cells in vitro and in vivo.

## 6. Conclusion

In recent years, over 500 articles regarding the mechanistic role of lncRNAs in anticancer RT have been published, and quite a part of them are about BRCA. These studies appear to open a door for us to regulate the radioresistance of BRCA through lncRNAs. We systematically reviewed the origins, classification, mechanisms of lncRNAs, the molecular mechanism of BRCA radioresistance, and the relationship between lncRNA and the radioresisitance of BRCA. Also, we screened the existing lncRNAs related to BRCA radioresistance one by one and summarized their mechanisms of action or signaling pathways, respectively. According to the research results, lncRNAs have been steadily becoming one of the current trends in regulating the radioresistance of BRCA. However, there are no clear guidelines based on any lncRNA in clinical practice to differentiate the BRCA patients who respond to RT from those who do not, at least for now. We believe that with the development of high-throughput sequencing technology, gene array technology, and bioinformatics analysis, an increasing number of potential lncRNAs related to BRCA radioresistance will be discovered. Additionally, we can predict the therapeutic effect and prognosis of cancer patients by monitoring the related signaling pathways, aiming to carry out more effective precise treatment and individualized treatment to improve the therapeutic efficacy of anti-BRCA RT.

## 7. Future Perspective

Several issues should be considered in investigating lncRNA as a regulatory factor of BRCA radioresistance. First, mammalian genomes are pervasively transcribed to produce thousands of lncRNAs, but only a few of them are confirmed to be associated with the radiosensitivity of BRCA at present. More efforts should be done to screen more lncRNAs related to BRCA radiosensitivity from the known lncRNAs or to discover new lncRNAs related to BRCA radioresistance from the unknown fields. Second, research in this field is in the embryonic or accelerating stage, and more validation is needed to verify the reliability of candidate lncRNAs as the regulators of BRCA radioresistance. Furthermore, lncRNAs have just been introduced into research on BRCA radioresistance; thus, lots of underlying intricate mechanisms remain to be clarified. Eventually, for those confirmed lncRNAs, specific gene silencing or enhancement technology can also be employed to improve the radiosensitivity of radioresistant BRCA, thus enhancing the therapeutic efficacy of RT for BRCA. Some researchers have reversed the radioresistance of BRCA by silencing lncRNA, which significantly enhance the radiotherapy effect of TNBC animal models [83]. Finally, it is expected that the profound implication of lncRNAs in anti-BRCA RT will be translated into clinical treatment through a series of further studies.

## Figures and Tables

**Table 1:** Classification of long non-coding RNAs into classes and sub-classes according to their function, structure and biogenesis.

lncRNAs		
Classification standards	Classes	Sub-classes
Function	Cis lncRNA	
	CeRNA	
	Trans lncRNA	

Structure	Circular RNA	
	Linear RNA	lincRNA
		eRNA
		TUCRNA
		NAT

Biogenesis	Intronic RNA	
	Enhancer RNA	
	Promoter RNA	
	Antisense RNA	
	Sense RNA	
	Intergenic RNA	
	Bidirectional RNA	

Abbreviations: cis-lncRNA: cis-acting long non coding RNA, ceRNA: competing endogenous RNA, trans-lncRNA: trans-acting long non coding RNA, lincRNA: long intergenic noncoding RNA, eRNA: enhancer-derived RNA, TUCRNAs: transcribed ultraconserved RNAs, NAT: natural antisense transcript.

## Data Availability

All the literature mentioned in this review article is publicly available from PubMed.
